# Pkd1l1 as a molecular sensor of fluid flow during the breaking of left-right symmetry

**DOI:** 10.1186/2046-2530-1-S1-P68

**Published:** 2012-11-16

**Authors:** DT Grimes, KL Riley, S Field, SH Patel, J Keynton, H Hilton, A Greenfield, DP Norris

**Affiliations:** 1 MRC Harwell, UK

## 

In mammals, left-right symmetry is broken during development by motile cilia-driven fluid flow in the embryonic node. How this ‘nodal flow’ is sensed remains the subject of debate. In kidney, fluid flow/membrane stress is sensed by polycystin protein complexes composed of Pkd1 and Pkd2 which reside in primary cilia. Pkd2 also plays a role in determining left-right asymmetry but no role has been found for Pkd1. We have isolated the ENU mouse mutant *rks* which harbours a mutation in the *Pkd1*-related gene *Pkd1l1*. *Pkd1l1rks* mutants exhibit severe left-right defects, loss of asymmetric gene expression but normal node cilia structure and motility. These data reveal the functional relationships between Pkd1 and Pkd1l1 which appear to be split between kidney development and left-right asymmetry respectively. *Pkd2lrm4* mutants strongly phenocopy *Pkd1l1rks* embryos with severe left-right defects. Based on this, we hypothesise that Pkd1l1 and Pkd2 are both required for the sensation of nodal flow. In support, we find that Pkd1l1 localises to primary cilia in a Pkd2-dependent manner and that Pkd1l1 and Pkd2 can form physical associations. The *Pkd1l1rks* point mutation lies in an extracellular PKD domain, specifically implicating this domain in the establishment of left-right asymmetry. We are performing cell-based experiments to understand the mechanism of Pkd1l1 function as well as computational and biophysical assays to assess the nature of the PKD domain and how its structure and function are affected by the *Pkd1l1rks* mutation.

